# Idiopathic choroidal neovascular membrane in a young female

**DOI:** 10.4103/0974-620X.57314

**Published:** 2009

**Authors:** Saad Abdullah Waheeb, Mahmood Jameel Showail

**Affiliations:** Department of Ophthalmology, King Abdulaziz University Hospital, Jeddah, Saudi Arabia

**Keywords:** *Bevacizumab*, *choroid*, *choroidal neovascularization*, *idiopathic*

## Abstract

A case of idiopathic choroidal neovascular membrane (CNVM) is described in a 17-year-old female patient. On initial examination her vision was counting fingers at one meter in the left eye (OS) and Fluorescein angiography showed a well-defined hyperfluorescent area corresponding to the CNVM. Intravitreal bevacizumab was injected into OS, and at a five-week follow-up visit, visual acuity improved to 20/100 OS. This case is unusual, in that the CNVM developed in a young lady with no significant past medical history and with the absence of a choroidal or retinal pigment epithelial disease process that may be associated with a CNVM.

## Introduction

In patients aged 50 years or younger, choroidal neovascularization (CNV) develops secondary to predisposing conditions that include pathological myopia, angioid streak, trauma or inflammation (e.g. ocular histoplasmosis syndrome).[1] In a significant number of young patients with CNV, no apparent cause can be detected, constituting idiopathic CNV.[2] These membranes are usually unilateral and final visual outcomes are considered to be more favorable than CNV due to age-related macular degeneration (AMD).[3] Only a few reports exist in the literature describing idiopathic CNV in young patients.[4] Several antivascular endothelial growth factor (VEGF) agents are now being administered intravitreally for CNV. The results are promising, and the procedure is considered to be safe and well-tolerated in the management of idiopathic CNV.[5] We report an idiopathic subfoveal CNV in a 17-year-old female with no evidence of any predisposing factor, which responded favorably to intravitreal bevacizumab injection.

## Case Report

**Figure 1:**
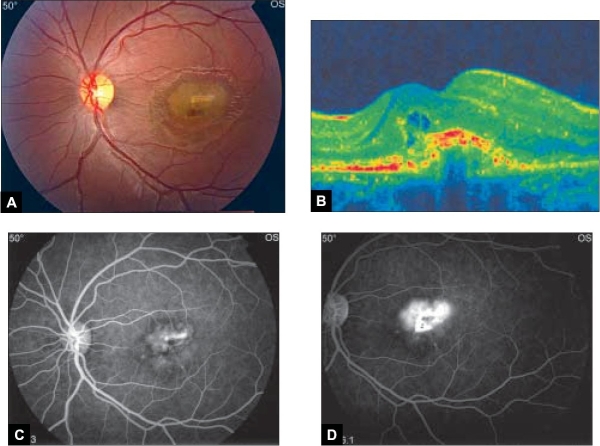
Left eye at presentation. (A) Color fundus photograph shows subfoveal choroidal neovascular membrane (CNVM) with subretinal heme, (B) transverse OCT shows intraretinal fluid that is consistent with an active CNV, (C) fundus fluorescein angiogram, early phase shows a well-defined hyperfluorescent area corresponding to the CNVM OS and (D) late phase shows diffuse leakage.

**Figure 2:**
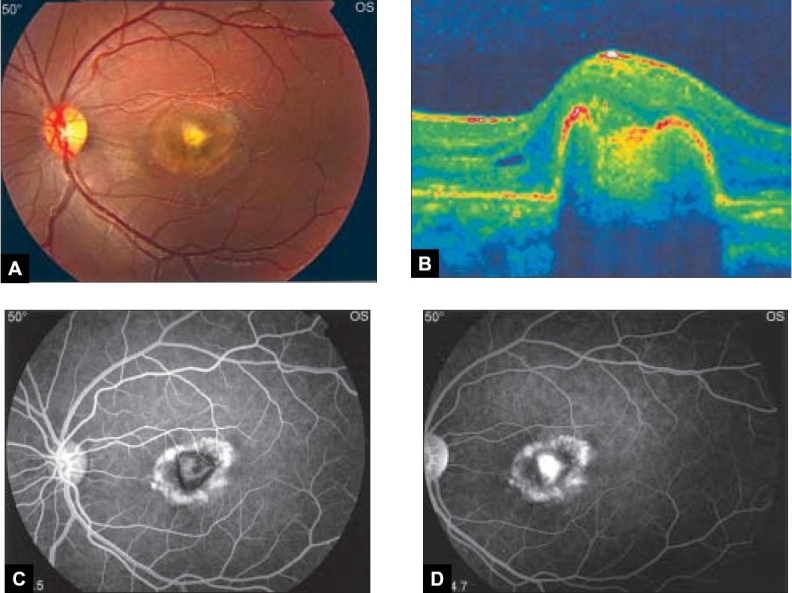
Left eye five weeks after single intravitreal bevacizumab injection. (A) Color fundus photograph shows disappearance of subretinal blood, (B) transverse OCT shows marked reduction in the intraretinal fluids, (C) fundus fluorescein angiogram, early phase shows contraction of the membrane and fluorescein dye staining of the CNV scar and (D) late phase show no further leakage

A 17-year-old high school female student was referred for evaluation of decreasing vision in her left eye (OS). There was no history of pain, redness or photophobia. There was no history of trauma. Past medical history was unremarkable. On examination, her best corrected visual acuity in the right eye (OD) was 20/20 and counting fingers at one meter OS. Her anterior segments were normal in both eyes (OU). On biomicroscopy of the posterior segments, subfoveal choroidal neovascular membrane (CNVM) with subretinal hemorrhage was noted OS [Figure 1A]. Fundus examination OD was unremarkable. No drusen, retinal pigment epithelial changes or macular exudates were observed OU. Fluorescein angiography [Figure 1C and D] showed a well-defined hyperfluorescent area corresponding to the CNVM OS with diffuse leakage on the late phase. Full blood investigations including serology were done to rule out any inflammatory pathology, and were normal. Chest X-ray was normal as well. After obtaining an informed consent, the left eye was anesthetized using topical benoxinate Hydrochloride 0.4%, then Intravitreal bevacizumab (Avastin; Genentech, Inc, South San Francisco, California) was injected into OS supratemporally 3.5 mm posterior to the limbus. At the five-week follow-up visit, visual acuity improved to 20/100 OS with marked reduction of fluid in optical coherence tomography scan (OCT) [Figure 2B]. Repeat fluorescein angiography showed contraction of the membrane and no further leakage in the late phase [Figure 2C and D]. The patient remains under follow-up

## Discussion

There are many etiologies of choroidal neovascularization (CNV), all of which are known or thought to occur with a defect in Bruch's membrane. To exclude the possibility of ongoing inflammation, a close biomicroscopic examination and full laboratory investigations were performed. However, there was no evidence of vitreous cells, condensation or vasculitis. There was no evidence of any systemic disease as well.

There are a few published studies on idiopathic CNV (case reports or small case series), which report variable visual results.[4‐6] Different treatment approaches for patients with CNV have been described: thermal laser therapy, ocular photodynamic therapy with verteporfin, transpupillary thermotherapy, submacular surgery and intravitreal injections of antivascular endothelial growth factor (VEGF) agents.[5,7‐10] As the natural history of Idiopathic CNV is better than that seen in AMD, thermal laser therapy does not appear to be a suitable treatment option because of the risk of immediate central vision diminution. Photodynamic therapy is too expensive and usually needs repeated sessions. Submacular surgeries are associated with a high risk of complications. Transpupillary thermotherapy is outdated and is widely replaced nowadays by anti-VEGF agents. The advantages of anti-VEGF agents are that they are available, easy to be administered and can be repeated as needed. With the introduction of anti-VEGF agents, numerous reports exist regarding their use as an off-label treatment of neovascular AMD.[11]

Several investigators have reported their observations following off-label treatment with bevacizumab in idiopathic CNV. Mandal *et al* reported results of intravitreal bevacizumab (1.25 mg/0.05 mL) in 32 eyes with idiopathic subfoveal CNV.[5] After 12 weeks follow-up, 19 eyes (59%) had an improvement in BCVA of three or more lines, 11 eyes (34%) remained stable and two eyes (6%) lost three or more lines. Their observations suggest that short-term use of intravitreal bevacizumab is safe and well tolerated in the management of idiopathic CNV.

Bevacizumab injection should be repeated if OCT shows intraretinal edema, subretinal fluid and/or pigment epithelial detachment at a four to six week interval. Although, intravitreal anti-VEGF injections appear to have a low complication rate, possible ocular complications include bacterial endophthalmitis, retinal detachments and uveitis.[12] Systemic side effects following intravitreal bevacizumab injection have been an area of debate. Acute elevation of blood pressure and stroke has been reported.[13] All these have to be made known to the patient and informed consent has to be obtained prior to therapy.

Ongoing studies monitoring ocular and systemic toxicities are vital in establishing the long-term safety profile of the anti-VEGF drugs. In our patient, the CNV regressed, intraretinal fluid was reabsorbed, and visual acuity improved significantly five weeks after VEGF injection and no adverse effect attributable to the drug or procedure were encountered in the follow-up period. However, our patient is under follow-up, and a re-injection would be performed if there is recurrence of edema. Obviously we cannot comment on the efficacy, limitations and long-term side effects of the treatment based on our case where a definite improvement of idiopathic CNV was observed following a single injection of bevacizumab over a five weeks follow-up period. We recommend a multicenter, prospective randomized controlled study to address the issue.
